# microRNA-26a shuttled by extracellular vesicles secreted from adipose-derived mesenchymal stem cells reduce neuronal damage through KLF9-mediated regulation of TRAF2/KLF2 axis

**DOI:** 10.1080/21623945.2021.1938829

**Published:** 2021-07-26

**Authors:** Zixin Hou, Ji Chen, Huan Yang, Xiaoling Hu, Fengrui Yang

**Affiliations:** a Department of Anesthesiology, The First Affiliated Hospital of University of South China, Hengyang, P. R. China; b Department of Endocrinology, The First Affiliated Hospital of University of South China, Hengyang, P. R. China

**Keywords:** Adipose-derived mesenchymal stem cells, extracellular vesicles, miR-26a, KLF9, TRAF2, KLF2, neuronal damage

## Abstract

Extracellular vesicles (EVs) are nano-sized vesicles secreted actively by numeorus cells and have fundamental roles in intercellular communication through shuttling functional RNAs. This study sets out to elucidate the role of microRNA-26a (miR-26a) shuttled by EVs derived from adipose-derived mesenchymal stem cells (ASCs) in neuronal damage. After extraction and identification of ASC-derived EVs (ASC-EVs), mouse cortical neuronal cells were selected to establish an in vivo cerebral ischemia/reperfusion mouse model and an in vitro oxygen glucose deprivation/reperfusion (OGD/RP) cell model. The downstream genes of miR-26a were analyzed. The gain- and loss-of function of miR-26a and KLF9 was performed in mouse and cell models. Neuronal cells were subjected to co-culture with ASC-EVs and biological behaviors were detected by flow cytometry, Motic Images Plus, TTC, TUNEL staining, qRT-PCR and western blot analysis. ASC-EVs protected neuronal cells against neuronal damage following cerebral ischemia/reperfusion, which was related to transfer of miR-26a into neuronal cells. In neuronal cells, miR-26a targeted KLF9. KLF9 could suppress the expression of TRAF2 and KLF2 to facilitate neuronal damage. In vitro and in vivo results showed that miR-26a delivered by ASC-EVs inhibited neuronal damage. In summary, ASC-EVs-derived miR-26a can arrest neuronal damage by disrupting the KLF9-meidated suppression on TRAF2/KLF2 axis.

## Introduction

Extracellular vesicles (EVs) are membrane vesicles that are released into the surrounding extracellular environment and can be divided into the subgroups microvesicles and exosomes [1,2]. EVs are heterogeneous small vesicles surrounded by a phospholipid bilayer and are secreted by virtually all cell types and are found in various biological fluids (blood, urine, saliva, cerebrospinal fluid, breast milk, etc.). EVs are rich in heat shock proteins HSP70 and HSP90, as well as endosome-specific proteins such as apoptosis-linked gene 2 interacting protein X (ALIX) and tumour susceptibility gene 101 (TSG101). In addition, EVs also contain cholesterol, ceramide, integrin, and tetraspanins (CD9, CD63, and CD81), all of which are typical components of lipid rafts, a type of microdomains in the plasma membrane. Lipid rafts are rigid membrane domains that are involved in lipid and protein sorting during endocytosis [3–5]. EVs are thought to be the mechanism by which cells exchange proteins, lipids and genetic material which collectively facilitate intercellular communication [2]. Once released into the extracellular space, they can reach the circulatory system and function systemically, where they discharge cargo into recipient cells and replicate the role of the parent cell [6]. EVs interact with recipient cells via different mechanisms. When internalized, EVs can release proteins, lipids and also nucleic acids such as miRNAs and messenger RNAs (mRNAs) that are functionally active inside cells. EVs exert several functions depending on the cell they originate.

Numerous diseases have been reported to be related to neuronal damage, such as cardiac arrest [7], destructive and developmental disturbances [8], ceramic ischaemic stroke [9], and spinal cord injury [10]. Neuronal damage has been shown to be linked to microRNAs (miRNAs or miRs) delivered by EVs [11,12]. EVs may have a pathogenic effect and promote disease progression but, conversely, EVs might also be protective and prevent the development of the Rheumatic disease [6,13,14]. Adipose-derived mesenchymal stem cell (ASC)-derived EVs (ASC-EVs) has been found to have diverse functions. Previous research reveals that ASC-EVs induced *in vitro* vessel-like structure formation by human mesenchymal endothelial cells (hMECs) [15], and also affected nerve growth [16] and wound healing [17,18].

miRNAs regulate various cellular processes by interfering with protein expression or mRNA degradation, with extensive research having focused on the role they result in apoptosis [19]. EVs derived from hMECs also deliver miRNAs to regulate the apoptosis and differentiation of neurons in patients with spinal cord injury [11]. miR-26a delivered from glioma stem cells into microvessel endothelial cells (MVECs) *via* EVs could potentially provide an effective therapeutic strategy for glioma by controlling phosphatase and tensin homolog deleted on chromosome 10 (PTEN) and regulating the phosphatidylinositol 3-kinase/protein kinase B (PI3K-Akt) pathway [20]. The thyroid hormone receptor/Kruppel-like factor 9 (KLF9) regulatory axis plays an important role in multiple stages of the hepatocyte lineage and determining when stem cell regeneration or begin the process of differentiation [21]. Existing studies have confirmed that circular RNA protein tyrosine phosphatase receptor type A (circPTPRA) competitively inhibits the expression of miR-636, thereby upregulating the expression of KLF9, which leads to reduced proliferation of blood cells [22]. In addition, knockdown of KLF9 can promote long-range axon regeneration after optic nerve injury, and its interaction with c-Jun N-terminal kinase 3 (JNK3) can inhibit axon growth *in vitro* and regenerative failure occurring in the body [23]. Based on the above information, this study focuses on the role miR-26a delivered by ASC-EVs and KLF9 both play in neuronal injury.

## Materials and methods

### Ethics statement

This study was approved by the Ethics Committee of the First Affiliated Hospital of University of South China and strictly performed according to the Guide for the Care and Use of Laboratory Animals published by the US National Institutes of Health. Extensive efforts were made to ensure minimal suffering of the included animals.

### Bioinformatics analysis

Downstream regulatory genes of miR-26a were predicted using the microT (http://diana.imis.athena-innovation.gr/DianaTools/index.php?r=microT_CDS/Index), miRanda (http://www.microrna.org/microrna/home.do) and RNAInter databases (http://www.rna-society.org/rnainter/). The intersection of the prediction results produced by the three databases was analysed using jvenn (http://jvenn.toulouse.inra.fr/app/example.htmL). Neuronal damage-related genes were searched with the ‘neuronal damage’ set as the keyword through the GeneCards database (https://www.genecards.org/), and then subjected to intersection analysis with the target genes of miR-26a using jvenn. In order to screen transcription factors from candidate target genes, we classified the types of proteins encoded by the genes using Panther (http://www.pantherdb.org/). To further predict the downstream regulatory factors of transcription factors, we used hTFtarget (http://bioinfo.life.hust.edu.cn/hTFtarget#!/) to retrieve the target genes of each transcription factor. Intersection analysis of the target gene and neuronal damage-related genes was conducted using jvenn. The intersected genes were subjected to Kyoto encyclopaedia of genes and genomes (KEGG) pathway enrichment analysis using R ‘clusterProfiler’ package with *p* < 0.05 as the threshold. Association analysis of enriched genes was performed using the STRING website (https://string-db.org/; minimum required interaction score = 0.9), and the results of interaction analysis were visualized through Cytoscape 3.5.1, with the genes at the core selected for further prediction.

### Animal model establishment

Eight-week-old male C57BL/6 mice were provided by the First Affiliated Hospital of University of South China. Middle cerebral artery occlusion (MCAO) was use to induce transient focal cerebral ischaemia. After intraperitoneal injection of 30 mg/kg sodium pentobarbital (Sigma–Aldrich Chemical Company, St. Louis, MO, USA) anaesthesia, 6–0 nylon monofilament suture was inserted into the right internal carotid artery to occlude the right middle cerebral artery (MCA). Mice were maintained at 37°C with a heated blanket throughout this procedure. Laser Doppler flowmeters was ensured successful occlusion (75–90% reduction in blood flow in the MCA area). 1 h after occlusion, reperfusion was performed by removing the suture. In the sham operation group, the same operation was performed without MCAO. Before MCAO surgery, EVs (100 mmol/kg/d, each mouse was injected at a dose of 100 mmol per kilogram per day) or 20 μL lentivirus overexpression vector was injected into the mouse lateral ventricle for 3 consecutive days. The mice were euthanized 72 h after reperfusion.

### Isolation and identification of ASCs

White adipose tissue samples from normal C57BL/6 male mice were minced with a surgical scalpel, and digested with 0.1% collagenase A (Roche Diagnostics GmbH Mannheim, Germany) in phosphate-buffered saline (PBS) 1% bovine serum albumin (BSA; Roche Diagnostics GmbH Mannheim, Germany) continuously at 37°C for 45 min. After Ficoll density separation (Lymphoprep; Axis-Shield, Oslo, Norway), cells were seeded at a density of 100,000 cells/cm^2^. Unattached cells were removed after 4 days and the medium was replaced with fresh medium. ASCs were cultured and enriched in Dulbecco’s modified Eagle’s medium (DMEM) (Gibco, Thermo Fisher Scientific Inc., Waltham, MA, USA) containing 100 U/mL penicillin, 100 μg/mL streptomycin (Gibco, Bleiswijk, the Netherlands), and 10% foetal bovine serum (FBS), at 37°C and 5% CO_2_. Flow cytometry was used to detect surface markers expressed on ASCs. Briefly, ASCs were trypsinized into single-cell suspensions. Cells were washed with PBS (excluding calcium and magnesium), blocked with 10% normal goat serum to prevent non-specific binding, and cultured with series of monoclonal antibodies: CD14, CD19, CD105, CD34, CD44, CD45, CD73, CD90, and human leukocyte antigen D-related (HLA-DR) (1:100, BioLegend, San Diego, CA, USA) labelled with fluorescein isothiocyanate (FITC) dyes for 30 min. A FITC-IgG isotype control was used in parallel. Next, cells were resuspended in 10% normal goat serum, and analysed using CyAn ADP Analyser (Beckman Coulter, Brea, CA, USA). Tri-lineage differentiation was performed in both cell types and confirmed by alkaline phosphatase (ALP) staining for osteogenic differentiation, Oil red O staining for adipogenic differentiation and Alcain blue staining for chondrogenic differentiation [24–27].

### Extraction and identification of ASC-EVs

EVs were extracted from ASCs (approximately 3.2 × 10^7^ cells) and passaged 2–3 times. When reaching about 70% confluence, ASCs were continued to be incubated in DMEM (including EV-removed FBS; to avoid contamination by EVs derived from FBS, the FBS was centrifuged at 1,100,000 × g for 2 h and the pellets were discarded) for 24–48 h. The EVs were extracted according to a previously reported methodology [28]. In brief, the ASC conditioned medium was subjected to gradient centrifugation and the supernatant was centrifuged at 70,000 × g for 1 h at 4°C. The debris particles contained in the EVs were washed with PBS, and centrifuged at 70,000 × g for 1 h. All ultracentrifugation steps were performed at 4°C in a Beckman ultracentrifuge (Optima L-90 K) with SW-32Ti rotor, resuspended in 200 µL PBS, and stored at −80°C. Hitachi H-7650 transmission electron microscope (TEM, Hitachi, Tokyo, Japan) was used to observe the EVs morphology. The distribution and particle size of EVs were measured using a Nanosizer™ instrument (Malvern Instruments, Malvern, UK) by dynamic light scattering (DLS). EV characteristics were identified by detection of specific surface markers CD63 (ab216130, 1:2000, Abcam Inc., Cambridge, UK, rabbit), TSG101 (ab125011, 1:10,000, Abcam, rabbit), CD81 (ab109201, 1:10,000, Abcam, rabbit), and Calnexin (ab92573, 1:100,000, Abcam, rabbit) using western blot analysis.

### Extraction of neuronal cells

Mouse primary cortical neuronal cells were obtained from neonatal mice. Mouse brain tissues were minced and incubated in 0.125% trypsin for 30 min. The reaction was stopped with DMEM/F12 medium containing FBS. The cell suspension was filtered and centrifuged (3000 × g, 10 min), and then the cell pellet was resuspended in DMEM/F12 medium. The cells were seeded on a 96-well plate coated with 10 mg/L poly-L-lysine (Sigma–Aldrich) at a density of 1 × 10^6^ cells/mL. After 72 h, arabinosylcytosine (5 µg/mL) (Shanghai Yuanye Biotechnology Co., Ltd.) was added to the cell culture medium to prevent the growth of non-neuronal cells. After 24 h, normal medium was used and the medium was changed every 72 h from that point onwards. Immunofluorescence was used to detect microtubule-associated protein 2 (MAP2) expression (A17409, 1:200, anti-rabbit, ABclonal Technology, Inc., USA), followed by identification of neuronal cells and purity.

### Establishing a of oxygen glucose deprivation/reperfusion (OGD/RP) neuronal cell model

OGD/RP method was used to treat neuronal cells. Briefly, cortical neuronal cells were exposed to glucose-free Earl’s solution supplemented with 5.4 mmoL/L KCl, 116.4 mmoL/L NaCl, 0.8 mmoL/L MgSO_4_, 1.8 mmoL/L CaCl_2_, 26.2 mmoL/L NaHCO_3_, 2.6 mmol/L NaH_2_PO_4_ and 20.1 mmol/L N-2-hydroxyethyl-piperazine-N’-2-ethanesulfonic acid (HEPES) (pH 7.4), and cultured at 37°C in 5% CO_2_ and 95% N_2_ (OGD) for 2 h. The reaction was terminated with glucose-free Earl’s solution containing 5.6 mmol/L glucose. Cell culture was continued for 12 h in an environment of 5% CO_2_ and 95% O_2_. Cortical neuronal cells were then treated with control PBS or EVs for 6 h and then with standard medium. After 72 h of treatment, cells received OGD/RP treatment.

### EV internalization

Purified EVs were labelled with the green fluorescent dye PKH67 (Sigma–Aldrich) as per the manufacturer instructions. Neuronal cells were seeded into 8-well chamber slides (Thermo Fisher Scientific) (8000 cells/well), and then 5 μL PKH67 was added to wells and incubated for 24 h to internalize EVs. Next, the mixture was incubated by shaking at 100 rpm and 4°C in the dark for 1 h with ice packs, followed by centrifugation at 1500 × g to remove the fluorescent dye PKH67 that had reacted with the EVs. Thereafter, 20 μL supernatant containing free fluorescent dye that had not reacted with the EVs was mixed with the internal standard (5 μL of 20 μM, 4 μM fluorescein in the sample solution) and was used for samples that were directly introduced into a capillary using a siphon method. Removing EVs before the internal standard addition was important because the internal standard would be adsorbed onto the EVs [29]. Finally, the slides were washed twice with PBS, fixed with 4% paraformaldehyde (Beijing Regen, Beijing, China) for 15 min, and nuclei stained with 4ʹ,6-diamidino-2-phenylindole (DAPI) (0.5 mg/mL; Invitrogen Inc., Carlsbad, CA, USA). Images were taken using a Zeiss LSM 780 (Zeiss, Jena, Germany) confocal microscope.

### Determination of infarct area

We anaesthetized the treated mice (eight mice per group). The brain was quickly excised and divided into six consecutive sections (± 5 mm, ± 3 mm and ± 1 mm from the pontine). Sections were stained at 37°C for 15 min using 2% 2,3,5-triphenyltetrazolium chloride (TTC, Sigma–Aldrich), and then fixed using 4% formaldehyde solution. A digital camera (Kodak DC240, East-man Kodak Co, USA) was used to photograph the infarcted area in each brain tissue slice. The infarct volume was calculated according to the following formula: lesion area per section = (area of contralateral hemisphere/area of ipsilateral hemisphere) × area of ipsilateral lesion. The volume of the lesion was estimated by multiplying the area of the lesion by the sum of the thickness of the slices.

### Immunofluorescence of NeuN

Frozen sections of mouse cerebral cortex and hippocampus were pre-incubated in PBS containing 0.3% Triton X-100 for 10 min, and blocked with 1% BSA, 0.1% Triton X-100 for 1 h. The primary and secondary antibodies used for immunofluorescence were rabbit anti-NeuN (ab177487, 1:500, rabbit anti, Abcam) and goat anti-rabbit IgG (AS011, abclone, 1:100), respectively. Nuclei were stained with a fixed medium containing DAPI, and then observed under a fluorescent inverted microscope (Olympus IX71, Olympus, Tokyo, Japan). Five sections of the cerebral cortex and the hippocampal CA1 area were calculated for each section, and NeuN positive cells counted using Image J software.

### Terminal deoxynucleotidyl transferase-mediated dUTP-biotin nick end labelling (TUNEL) Staining

Apoptosis in the cerebral cortex was examined using a TUNEL apoptosis analysis kit (Beyotime Institute of Biotechnology, Shanghai, China) according to the manufactures protocol. In short, 72 h after MCAO, frozen sections of brain tissue specimens were obtained, fixed with 4% paraformaldehyde for 30–60 min, and washed twice with PBS for 10 min. Brain sections were added with Immunostaining Power Permeate (P0097) or PBS containing 0.3% Triton X-100 and incubated for 5 min at room temperature. To inactivate the endogenous peroxidase, the sections were incubated with endogenous peroxidase blocking solution (P0099) (Beyotime) or 0.3% H_2_O_2_ in PBS at room temperature for 20 min. TUNEL detection solution (50 μL) was added to the sample and incubated in the dark at 37°C for 60 min. The samples were washed once with PBS, added with 0.1–0.3 mL of the labelling reaction termination solution and incubated at room temperature for 10 min. Following three washes with PBS, the samples were incubated with 50 μL of streptavidin-horseradish peroxidase (HRP) working solution at room temperature for 30 min. Next, 0.2–0.5 mL of 3,3ʹ-diaminobenzidine tetrahydrochloride (DAB) solution was added to the samples and incubated at room temperature for 5–30 min. The samples were counterstained with haematoxylin, mounted and observed.

### Cell transfection

Neuronal cells or ASCs were treated with lentiviral vector pLVX-miR-26a mimic/inhibitor (Ambion, Carlsbad, CA) and lentiviral packaging overexpression plasmids (overexpression [oe]-KLF9, oe-tumour necrosis factor receptor (TNFR)-associated factor 2 [TRAF2], and oe-KLF2) (GeneChem, Shanghai, China). *In vitro* experiments: 1) control: OGD/RP, OGD/RP + PBS, OGD/RP + ASC-EVs, 2) inhibitor-negative control (NC), miR-26a inhibitor, mimic-NC, miR-26a mimic, 3) oe-NC + PBS, oe-KLF9 + PBS, oe-NC + ASC-EVs, oe-KLF9 + ASC-EVs, 4) oe-KLF9, oe-TRAF2 + oe-KLF9, oe-TRAF2, oe-NC, 5) EVs-inhibitor-NC + oe-NC, EVs-inhibitor-NC + oe-KLF2, EVs-miR-26a inhibitor + oe-NC, EVs-miR-26a inhibitor + oe-KLF2.

*In vivo* experiments: 1) sham, MCAO, MCAO + PBS, MCAO + ASC-EVs, 2) EVs-inhibitor-NC + oe-KLF2, EVs-miR-26a inhibitor + oe-KLF2, EVs-inhibitor-NC + oe-NC, EVs-miR-26a inhibitor + oe-NC.

### Detecting cellular apoptosis

Apoptosis of cortical neuronal cells was detected by flow cytometry (Beckman-Coulter, Brea, CA) using the Annexin V-FITC/PI kit (BD PharMingen, San Diego, CA). Briefly, after OGD treatment cells were seeded in 24-well plates at a density of 1 × 10^6^ cells/well. The cells were incubated with Annexin V/CY3 for 15 min at room temperature. After washing, the cells were added with propidium iodide (PI) and incubated for 30 min, after which the rate of apoptosis was determined.

### Determination of neurite outgrowth length

Neuronal cells were inoculated on a thin film petri dish (FD10300, Matsunami Glass, Ltd., Osaka, Japan). In order to determine the neurite outgrowth length, Motic Images Plus (Motica China Group Co., Ltd., Fujian, China) was used to calculate the total neurite outgrowth length by adding all protrusion lengths measured on a single neuron. At least five neuronal cells were randomly selected from each treatment group for evaluation.

### Dual luciferase reporter assay

A mouse KLF9 3ʹ-untranslated region (3ʹ-UTR) sequence or a KLF9 3ʹ-UTR mutant sequence containing a predicted miR-26a binding site was inserted into a pGL3 promoter vector (Genscript, Nanjing, China). The HEK293T cell line (American Type Culture Collection, Manassas, VA, USA) was seeded in a 24-well plate at a density of 5 × 10^5^ cells/well one day before transfection. Subsequently, luciferase reporter vector (0.12 μg) and miR-26a mimic or mimic NC were co-transfected into the cells using Lipofectamine 3000 transfection reagent (Invitrogen). The experimental results were analysed 48 h after transfection.

### Chromatin immunoprecipitation (ChIP)

ChIP was used to verify the binding of KLF9 to the TRAF2 promoter region using a ChIP detection kit (Millipore corp., Billerica, MA, USA). The cells were cross-linked with 1% formaldehyde, washed and resuspended in SDS lysis buffer. The nuclei were then ultrasonicated. Chromatin components were removed with protein A-agarose beads after which the cells were then incubated with KLF9 antibodies (A7196, 1:2000, rabbit, ABclonal) or corresponding control antibodies (anti-rabbit IgG, ab171870, Abcam; anti-mouse IgG1, ab81032, Abcam). Crosslinking was cleaved and digestion with proteinase K was conducted. Amplification and quantification of the immunoprecipitated DNA were performed using quantitative reverse transcription-polymerase chain reaction (qRT-PCR).

### qRT-PCR

Total RNA was extracted from cells using TRIzol reagent (Invitrogen). For mRNA detection, 1 μg total RNA was reverse transcribed into complementary DNA (cDNA) using the Revert Aid first-strand cDNA synthesis kit (Fermentas, Life Sciences, Canada). qRT-PCR analysis was performed using SYBR Premix ExTaq^TM^II in an ABI PRISM® 7900HT System (Takara Biotechnology, Japan). With glyceraldehyde-3-phosphate dehydrogenase (GAPDH) serving as the internal reference gene, the relative mRNA expression was determined using the relative standard curve method (2 ^−ΔΔCT^). PCR primers are shown in Table S1. miRNA analysis was performed using the SeraMir Evssome RNA Purification Kit (System Biosciences, Mountain View, USA) to isolate EV miRNA. The miRNA cDNA was synthesized according to the instructions of TaqMan microRNA assay kit (Applied Biosystems, Foster City, USA). For qRT-PCR reactions, universal reverse primers provided by FastStart Universal SYBR Green Master Mix (Roche, Indianapolis, USA) with the miRNA-specific forward primer (Sangon Biotech, Shanghai, China) and TaqMan microRNA assay kit were used. Results were normalized using Cel-miR-39 small nuclear RNA.

### Western blot analysis

Total protein content was separated using sodium dodecyl sulphate-polyacrylamide gel electrophoresis (SDS-PAGE) and transferred onto polyvinylidene fluoride membranes (Immobilon P, Millipore, Billerica, USA). After blocking with 5% milk and 0.1% Tween-20 Tris-buffered saline at room temperature for 1 h, the membrane was incubated overnight at 4°C with primary rabbit antibodies against KLF9 (A7196, 33kd, 1:2000, ABclonal), TRAF2 (A0962, 53kd, 1:2000, ABclonal), KLF2 (A16480, 31kd, 1:2000, ABclonal), caspase-3 (A2156, 37 kDa, 1:2000, ABclonal), cleaved caspase-3 (ab32042, 17kd, 1:500, Abcam), Bcl-2-associated X protein (Bax) (A0207, 21 kDa, 1:2000, ABclonal), B-cell lymphoma 2 (Bcl-2) (A0208, 26 kDa, 1:2000, ABclonal), nerve growth factor (NGF) (A17998, 27 kDa, 1:2000, ABclonal), and neurite growth inhibitory factor-A (NOGO-A) (BA1309, 130 kDa, 1:2000, Boster Biological Technology Co., Ltd., Wuhan, Hubei, China). After three washes with Tris-buffered saline Tween-20 (TBST), the membrane was incubated with HRP-conjugated secondary antibody anti-rabbit IgG (AS014, 1:10,000, ABclonal) and anti-mouse IgG (AS003, 1:10,000, ABclonal) at 37°C for 1 h. Immunoreactive bands were visualized using enhanced chemiluminescence reagent (Thermo Fisher Scientific) and imaged using ChemiDoc XRS Plus luminescent image analyser (Bio-Rad Laboratories, Hercules, CA, USA). Image-Pro Plus 6.0 software was used to quantify the band intensity (3 replicates), and the relative expression of the target protein was normalized using the band intensity of GAPDH (AC033, 1:50,000, ABclonal).

### Statistical analysis

SPSS 21.0 statistical software (IBM Corp., Armonk, NY, USA) was used for data statistical analysis. The measurement data was expressed as mean ± standard deviation throughout this study. The data between the two groups were compared using unpaired *t-*test. Data comparison among multiple groups was performed using one-way analysis of variance (ANOVA) followed by Tukey’s post hoc tests with corrections for multiple comparisons. In all statistical analysis, a value of *p* < 0.05 represents statistical significance.

## Results

### ASC-EVs relieve neuronal damage after cerebral ischaemia and reperfusion

**Figure 1.**
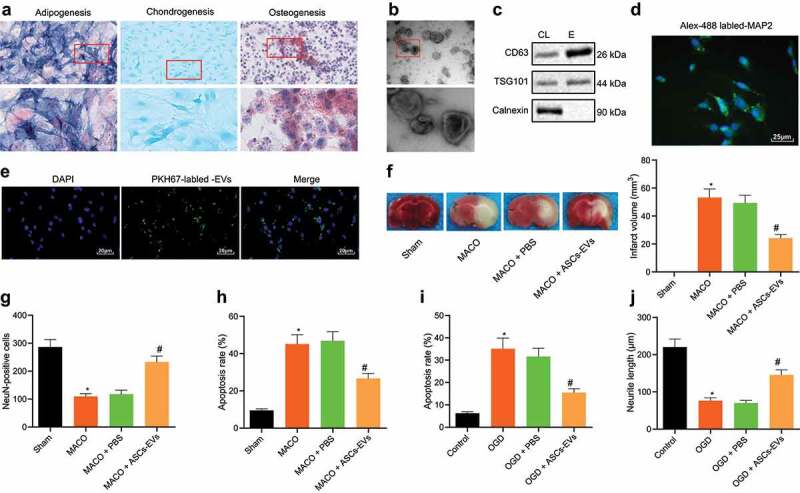
ASC-EVs protect neuronal cells against cerebral ischaemia/reperfusion

ASC-EVs have been shown to promote the growth of neurites after crush injury and thereby reduce neuronal damage [30]. In order to elucidate the mechanism by which ASCs act during neuronal injury in cerebral ischaemia/reperfusion we initially extracted and characterized ASCs. Surface markers of ASCs were identified by flow cytometry. The results showed that CD29 (99%), CD44 (99%), CD73 (100%), CD90 (99%), CD105 (99%) and CD166 (99.8%) were highly expressed, while CD14 (5%), CD31 (1.5%) and CD45 (4.5%) were poorly expressed (Figure S1A). In addition, the cells had osteogenesis, adipogenic and chondrogenic differentiation ability (Figure 1a). These results indicated that ASCs had been successfully isolated using this approach.

A: Osteogenic, adipogenic and chondrogenic differentiation of ASCs determined by ALP (200 ×), Oil red O staining (400 ×), and Alcain blue staining (400 ×), respectively. B: Observation of EV morphology under a TEM. Scale bar = 100 nm. C: Western blot analysis of EV specific surface marker proteins in EVs. D: Immunofluorescence analysis of MAP2 expression in mouse primary cortical neuronal cells (400 ×). E: Fluorescence microscope analysis of the internalization of EVs by neuronal cells following 24 h of incubation of PKH67-labelled EVs with neuronal cells (400 ×). F: TTC staining of cerebral infarct area in brain tissues of MCAO mice treated with ASC-EVs. G: NeuN immunofluorescence staining of living neuronal cells in brain tissues of MCAO mice treated with ASC-EVs. H: TUNEL staining of cell apoptosis in MCAO mouse brain tissues. I: Flow cytometric analysis of apoptosis of OGD-induced neuronal cells treated with ASC-EVs. J: The neurite outgrowth length of OGD-induced neuronal cells treated with ASC-EVs. *, *p* < 0.05, *vs*. the sham-operated mice or control cells; #, *p* < 0.05, *vs*. MACO mice treated with PBS or OGD-induced neuronal cells treated with PBS. n = 8 for mice in each group. The measurement data were expressed as mean ± standard deviation of at least three samples. Data comparison among multiple groups was performed using one-way ANOVA and Tukey’s post hoc test. Cell experiments were repeated three times independently.

Examination of the EVs released from ASCs using TEM microscopy showed that EVs had a cup-like or spherical shape (Figure 1b). DLS test results showed that the diameter of EVs was mainly distributed around 100 nm (Figure S1B). Moreover, study of EV surface markers by western blot analysis revealed that CD63 and TSG101 expressions were higher in EVs compared to cell debris (Figure 1c). These results demonstrated that we could reliably isolate EVs. At the same time, immunofluorescence data on the fluorescence-activated cell sorting (FACS) analysis of MAP2 in mouse primary cortical neuronal cells displayed the successful neuron extraction (Figure 1d) and the corresponding quantitative analysis results suggested that the purity was above 95% (Figure S1C). Neuronal cells were therefore incubated with ASC-EVs for 24 h, and fluorescence microscopy showed that neuronal cells can internalize ASC-EVs (Figure 1e).

In order to further study the function of EVs in this setting, we injected PBS or ASC-EVs (100 mmol/kg/d) into the lateral ventricle of the mouse for 3 consecutive days, and performed MCAO surgery. After 72 h of reperfusion, the mice were euthanized and brain tissue slices were taken from the mice. Analysis on the cerebral infarction using TTC staining indicated that ASC-EVs significantly reduced the area of cerebral infarction (figure 1f). In addition, NeuN immunofluorescence staining experiments (Figure 1g) revealed that ASC-EVs significantly increased the number of NeuN+ cells and the survival of neuronal cells. Meanwhile, western blot analysis was conducted to determine the EV intake in mouse brain tissues, the results of which presented that ASC-EVs significantly increased the number of NeuN+ cells and survival of neuronal cells, and the expression of CD63 and TSG101 was the highest in the brain tissues (Figure S1D). The results of TUNEL staining (Figure 1h) also displayed that ASC-EVs significantly reduced the number of apoptotic cells. After incubating neuronal cells with ASC-EVs, and followed by OGD/RP treatment, ASC-EVs significantly reduced the apoptosis rate of cells after OGD/RP treatment (Figure 1i) while also reducing neurite outgrowth damage (Figure 1j). It therefore appears that ASC-EVs slow the damage of neuronal cells caused by cerebral ischaemia/reperfusion.

### ASC-EV miRNA miR-26a recapitulates this reduction in neuronal damage

**Figure 2.**
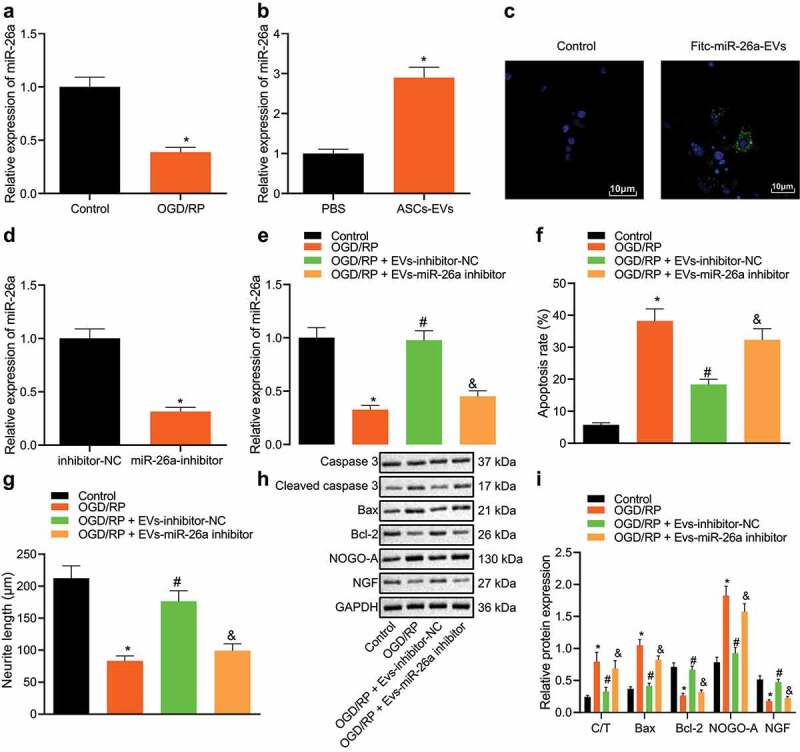
miR-26a is secreted by ASCs EVs and reduces neuronal damage

miR-26a has been reported to prevent apoptosis of neural stem cells in brain injuries caused by cardiac arrest [31]. Moreover, ASC-EVs have been shown to contain large amounts of miR-26a [32]. We thus hypothesized that ASC-EVs could reduce neuronal apoptosis by transferring miR-26a. The results of qRT-PCR suggested that after OGD/RP treatment, the expression of miR-26a in neuronal cells was significantly reduced (Figure 2a). Additionally, ASC-EVs significantly increased the expression of miR-26a in neuronal cells (Figure 2b). In order to confirm that miR-26a was transferred to neuronal cells through ASC-EVs, FITC labelled miR-26a mimic was transfected into ASCs. EVs were then extracted and incubated with neuronal cells, after which a clear green fluorescence was identified in the treated neuronal cells (Figure 2c).

A: miR-26a expression in neuronal cells determined by qRT-PCR in neuronal cells after OGD/RP treatment. *, *p* < 0.05, *vs*. control cells. B: miR-26a expression in ASC-EVs treated neuronal cells determined by qRT-PCR. *, *p* < 0.05, *vs*. PBS treated neuronal cells. C: The entry of FITC-labelled miR-26a mimic into neuronal cells determined using a fluorescence microscope (scale bar = 25 μm). D: miR-26a expression in miR-26a inhibitor-treated ASCs determined by qRT-PCR. *, *p* < 0.05 *vs*. inhibitor-NC treated neuronal cells. E: The expression of miR-26a determined by qRT-PCR in neuronal cells treated with EVs-miR-26a inhibitor. F: Apoptosis of OGD/RP-induced neuronal cells treated with EVs-miR-26a inhibitor detected by flow cytometry. G: Neurite length in OGD/RP-induced neuronal cells treated with EVs-miR-26a inhibitor measured by Motic Images Plus. H, I: The expression of apoptosis-related proteins caspase-3, cleaved caspase-3, Bax, Bcl-2, NGF and NOGO-A determined by western blot analysis in OGD/RP-induced neuronal cells treated with a EVs-miR-26a inhibitor. C/T represents the ratio of cleaved caspase-3/caspase-3. (E-I) *, *vs*. control cells, #, *vs*. OGD/RP treated neuronal cells, and &, *vs*. OGD/RP + EVs-inhibitor-NC treated neuronal cells, *p* < 0.05. The measurement data were expressed as mean ± standard deviation. In panel A, B, D and E, data were compared by unpaired *t-*test; in panel F, G, and I, data were compared by one-way ANOVA and Tukey’s post hoc test. The experiment was repeated three times independently.

To further verify the role of ASC-EVs-miR-26a in neuronal damage, ASCs were treated with a miR-26a inhibitor, which successfully significantly reduced miR-26a expression in ASCs (Figure 2d). Next, EVs were extracted and neuronal cells were treated. We found that compared with the EVs-inhibitor NC treated neuronal cells, the expression of miR-26a in the EVs-miR-26a inhibitor treated neuronal cells was significantly reduced (Figure 2e). After OGD/RP treatment, when compared with the RP + EVs-inhibitor-NC, the apoptosis of OGD/RP + EVs-miR-26a inhibitor treated neuronal cells was increased (figure 2f), the length of neurite outgrowth was reduced (Figure 2g), cleaved caspase-3/caspase-3, Bax, and NOGO-A expression were enhanced, and the expression of Bcl-2 and NGF was inhibited (Figure 2(h, I)). Therefore, ASC-EVs contain a large number of miR-26a, which they deliver to neuronal cells through EVs, thus protect neuronal cells from damage.

### miR-26a targets KLF9 and inhibits its expression

**Figure 3.**
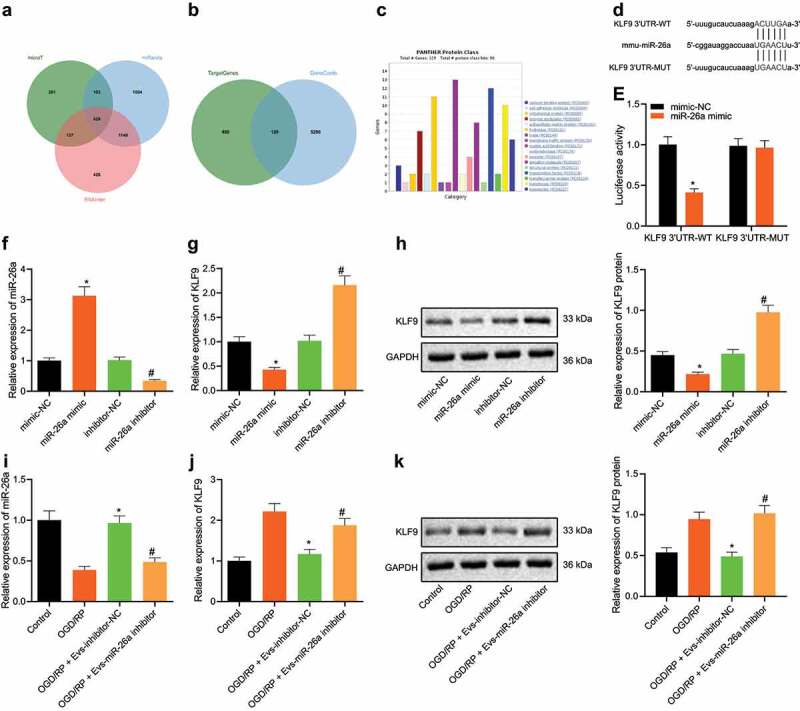
KLF9 is a direct target gene of miR-26a

In order to further study the molecular mechanism by which ASC-EVs miR-26a reduce neuronal damage, we first predicted the downstream regulatory genes of miR-26a using the microT, miRanda and RNAInter databases. The genes predicted in both datasets are summarized in the Venn Diagram in Figure 3a, with, 529 genes were found at the intersection (Figure 3a). In addition, intersection analysis of the predicted target genes and neuronal damage-related genes retrieved from GeneCards reduced this to 129 candidate genes (Figure 3b). Classification of the types of proteins encoded in genes by the Panther website showed that 12 of these genes act as transcription factors which regulate the molecular mechanisms involved (Figure 3c). A previous study confirmed that the knockout of KLF9, axon growth inhibitory factor, can promote the regeneration of neuron axons [24], and KLF9 may affect the repair of neuronal cells after injury. The TargetScan website predicted the presence of miR-26a binding sites in the 3ʹUTR of KLF9 (Figure 3d). Dual-luciferase reporter assay data further revealed that miR-26a mimic decreased the luciferase activity of KLF9-3ʹUTR-WT without altering that of KLF9-3ʹUTR-MUT (Figure 3e).

A: Venn diagram summarizing the downstream regulatory genes of miR-26a as predicted by the microT, miRanda and RNAInter databases. B: Venn diagram outlining the neuronal damage-related genes in GeneCards with 529 candidate target genes. C: Cluster analysis of the protein types encoded by genes in the Panther website. The abscissa indicates protein category, and the ordinate indicates number of genes in each category. D: Putative miR-26a binding sites in the 3ʹUTR of KLF9 mRNA in the miRanda website. E: miR-26a binds directly to KLF9, as confirmed by dual-luciferase reporter assay in HEK293T cells. F: miR-26a expression detected by qRT-PCR in neuronal cells transfected with miR-26a mimic and inhibitor. G: KLF9 mRNA expression detected by qRT-PCR in neuronal cells transfected with miR-26a inhibitor and a control mimic. H: KLF9 protein expression analysed by western blot analysis in neuronal cells transfected with miR-26a mimic and inhibitor. (E-H) *, *p* < 0.05 *vs*. mimic NC treated neuronal cells, #, *p* < 0.05 *vs*. inhibitor NC treated neuronal cells. I: The expression of miR-26a detected by qRT-PCR in ASC-EVs treated neuronal cells. J: The expression of KLF9 detected by qRT-PCR in neuronal cells after ASC-EV treatment. K: The expression of KLF9 detected by western blot analysis in neuronal cells after ASC-EV treatment. (I ~ K) *, *p* < 0.05 *vs*. OGD/RP treated neuronal cells. #, *p* < 0.05, *vs*. OGD/RP + EVs-inhibitor-NC treated neuronal cells. The measurement data were expressed as mean ± standard deviation. In panel E, data were compared by unpaired *t-*test, in panel F, G, H, I, J, and K, data were compared by one-way ANOVA and Tukey’s post hoc test. The experiment was repeated three times independently.

Next, after miR-26a mimic and inhibitor were transfected into neuronal cells, miR-26a expression was examined by qRT-PCR (figure 3f), and KLF9 expression was assessed by qRT-PCR (Figure 3g) and western blot analysis (Figure 3h). Results demonstrated that compared to mimic-NC, miR-26a expression was upregulated in miR-26a mimic treated neuronal cells, while KLF9 mRNA and protein levels were downregulated. Compared to inhibitor-NC, miR-26a expression was downregulated, but KLF9 mRNA and protein levels were upregulated in miR-26a mimic treated neuronal cells. At the same time, after treating neuronal cells with ASC-EVs, miR-26a expression was analysed by qRT-PCR (Figure 3i), KLF9 levels in neuronal cells by qRT-PCR (Figure 3j) and western blot analysis (Figure 3k). This demonstrated that compared with OGD/RP, OGD/RP + EVs-inhibitor-NC significantly increased the expression of miR-26a and decreased the expression of KLF9. In comparison with OGD/RP + EVs-inhibitor-NC, in OGD/RP + EVs-miR-26a inhibitor cells, miR-26a expression was downregulated while KLF9 expression was upregulated. Taken together, these results indicate that ASC-EVs-derived miR-26a can target and inhibit KLF9 expression in neuronal cells.

### miR-26a targets KLF9 to protect neuronal cells

**Figure 4.**
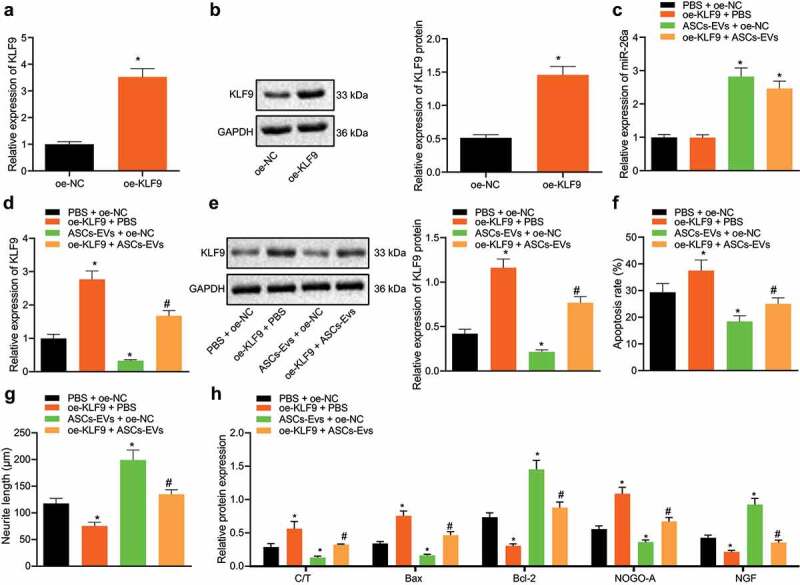
Overexpression of KLF9 reverses the protective effect ASC-EVs have on neuronal cells

We then aimed to verify the effect ASC-EVs miR-26a targeting of KLF9 has on neuronal cells. qRT-PCR and western blot analysis confirmed the overexpression efficiency of oe-KLF9 in neuronal cells, as shown by significantly increased KLF9 expression in the presence of oe-KLF9 (Figure 4(a, B)). After ASC-EV treatment and transfection with oe-KLF9, the functional changes of neuronal cells were observed after OGD/RP treatment. RT-PCR analysis revealed that miR-26a expression was enhanced following treatment with ASC-EVs (Figure 4c).

A: oe-KLF9 overexpression efficiency in neuronal cells confirmed by qRT-PCR. B: Overexpression efficiency of oe-KLF9 in neuronal cells confirmed by western blot analysis. C: miR-26a expression detected by qRT-PCR in neuronal cells following treatment with ASC-EVs, oe-KLF9 or both. D: KLF9 expression detected by qRT-PCR in neuronal cells treated with ASC-EVs, oe-KLF9 or both. E: KLF9 expression detected by western blot analysis of neuronal cells after treatment with ASC-EVs, oe-KLF9 or both. F: Apoptosis of neuronal cells after treatment with ASC-EVs, oe-KLF9 or both detected by flow cytometry. G: Length of neurite outgrowth as observed by Motic Images Plus. H: Expression of cleaved caspase-3, caspase-3, Bax, Bcl-2, NOGO-A, and NGF analysed detected by western blot analysis in neuronal cells treated with ASC-EVs, oe-KLF9 or both. *, *p* < 0.05 vs. oe-NC- or PBS + oe-NC treated neuronal cells, #, *p* < 0.05 vs. ASC-EVs + oe-NC treated neuronal cells. The measurement data were expressed as mean ± standard deviation. In panel A, and B, data were compared by unpaired *t-*test, and in panel C, D, E, F, G, and H, data were compared by one-way ANOVA and Tukey’s post hoc test. The experiment was repeated three times independently.

Subsequent results of qRT-PCR (Figure 4d) and western blot analysis (Figure 4e) suggested that, relative to PBS + oe-NC treatment, the expression of KLF9 was increased upon ASC-EVs + oe-NC treatment, while it was also increased following oe-KLF9 + PBS treatment. Compared with treatment with ASC-EVs + oe-NC, KLF9 expression was significantly increased in response to treatment with oe-KLF9 + ASC-EVs. In addition, compared to treatment with PBS + oe-NC, treatment with oe-KLF9 + PBS significantly promoted apoptosis and shortened the length of neurite outgrowth, while ASC-EVs + oe-NC treatment increased cell survival rate and the length of neurite outgrowth while reducing apoptosis. Cells treated with oe-KLF9 + ASC-EVs had increased apoptosis and shorter neurite outgrowth than those in cells treated with ASC-EVs + oe-NC (Figure 4(f, G)).

Western blot analysis results (Figure 4h) also showed that, when compared with treatment with PBS + oe-NC, ASC-EVs + oe-NC treatment inhibited the expression of Bax and NOGO-A and increased that of Bcl-2 and NGF, while treatment with oe-KLF9 + PBS produced the opposite effect. However, compared with treatment with ASC-EVs + oe-NC, treatment with oe-KLF9 + ASC-EVs promoted the expression of cleaved caspase-3/caspase-3, Bax and NOGO-A, and decreased that of Bcl-2 and NGF. Thus, it appears that overexpression of KLF9 upregulates numerous aspects of the apoptotic machinery, reversing the protective effect ASC-EVs have on neuronal cells.

### KLF9 regulates KLF2 expression by determining TRAF2 expression

**Figure 5.**
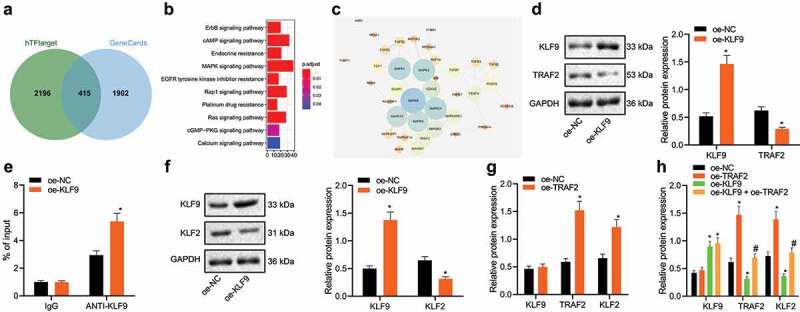
KLF9 downregulates TRAF2 and KLF2 expression thereby affecting neuronal damage

In order to further predict the downstream regulators of KLF9, we screened 415 genes to find the target genes of KLF9 through hTFtarget and going to identify any of these genes which have been linked to neuronal damage-related genes in GeneCards (Figure 5a). Enrichment of the 415 genes by the KEGG pathway revealed that 39 genes were enriched in the mitogen-activated protein kinase (MAPK) signalling pathway (Figure 5b). Further use of the STRING website to analyse the interaction of 39 genes allowed interaction analysis to be visualized, revealing that 12 genes were at the core position of the network map (Degree ≥ 7) (Figure 5c).

A: Venn diagram summarizing the intersection of the KLF9 target gene predicted by the biological website hTFtarget and the neuronal damage-related genes in GeneCards. B: KEGG pathway enrichment analysis of 415 the identified candidate genes. The abscissa indicates number of genes and the ordinate indicates signalling pathway name. C: Interaction analysis network of 39 genes constructed by the STRING. The circles from large to small indicates the degree value of the gene, the circle colour from blue to orange indicates the degree from large to small, and the line in the middle of the circle indicates the interaction relationship between genes. D: KLF9 and TRAF2 expression detected by western blot analysis in oe-KLF9 treated neuronal cells. E: The binding of KLF9 to the TRAF2 promoter region as determined using a ChIP-qPCR approach. F: KLF2 expression detected by western blot analysis in oe-KLF9 treated neuronal cells. G: KLF9, TRAF2, and KLF2 expression determined by western blot analysis in oe-TRAF2 treated neuronal cells. H: KLF9, TRAF2, and KLF2 expression detected by western blot analysis in neuronal cells treated with oe-KLF9, oe-TRAF2 or both. *, *p* < 0.05, *vs*. oe-NC treated neuronal cells, #, *p* < 0.05, *vs*. oe-KLF9 treated neuronal cells. The measurement data were expressed as mean ± standard deviation. In panel D, E, F, and G, data were compared by unpaired *t-*test, and in panel H, data were compared by one-way ANOVA and Tukey’s post hoc test. The experiment was repeated three times independently.

Analysis of KLF9 ChIP-seq data revealed that KLF9 binds to the TRAF2 promoter. The expression of KLF9 and TRAF2 in oe-KLF9 treated neuronal cells was detected by western blot analysis. Oe-KLF9 treated neuronal cells exhibited significantly upregulated expression of KLF9 and reduced expression of TRAF2, suggesting that KLF9 transcription inhibited the expression of TRAF2 in neuronal cells (Figure 5d). Perhaps unsurprisingly, ChIP-qPCR experiments also demonstrated that overexpression of KLF9 can increase the binding of KLF9 to the TRAF2 promoter region (Figure 5e). Moreover, western blot analysis results indicated that KLF9 overexpression in neuronal cells suppressed KLF2 expression (figure 5f), while overexpression of TRAF2 enhanced the expression of TRAF2 and KLF2 (Figure 5g). Western blot analysis data further revealed that oe-TRAF2 treated neuronal cells showed upregulated expression of TRAF2 and KLF2 (Figure 5h), and oe-KLF9 treated neuronal cells showed upregulated KLF9 expression and downregulated TRAF2 and KLF2 expression compared with oe-NC treated neuronal cells. However, the combined treatment with oe-TRAF2 and oe-KLF9 reversed the inhibiting effect of oe-KLF9 on the TRAF2 and KLF2 expressions. Therefore, it appears that KLF9 affects neuronal damage by regulating the expression of TRAF2 and KLF2

### KLF9 regulates the TRAF2/KLF2 axis to prevent neuronal damage

**Figure 6.**
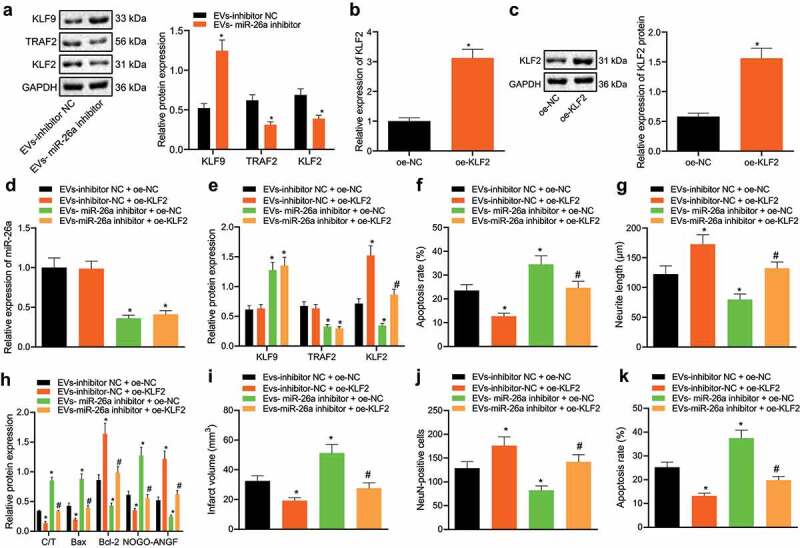
ASC-EVs miR-26a targeting KLF9 affects TRAF2/KLF2 regulatory axis to prevent neuronal damage

Next, we sought to further verify that ASC-EVs miR-26a regulates neuronal damage through the KLF9/TRAF2/KLF2 regulatory axis. The results of western blot analysis revealed that compared with EVs-inhibitor NC, EVs-miR-26a inhibitor increased the expression of KLF9 and inhibited that of TRAF2 and KLF2 (Figure 6a). Moreover, results of qRT-PCR (Figure 6b) and western blot analysis (Figure 6c) showed that oe-KLF2 can upregulate KLF2 expression. As shown in Figure 6(d-E), compared with EVs-inhibitor NC + oe-NC, EVs-miR-26a inhibitor + oe-NC reduced miR-26a, TRAF2, and KLF2 expressions, while increasing KLF9 expression, and EVs-inhibitor-NC + oe-KLF2 increased the expression of KLF2. However, compared to miR-26a inhibitor + oe-NC, miR-26a inhibitor + oe-KLF2 increased KLF2 expression.

A: The expression of KLF9, TRAF2, and KLF2 as determined by western blot analysis in EVs-inhibitor-NC and EVs-miR-26a inhibitor treated neuronal cells. *, *p* < 0.05 *vs*. EVs-inhibitor NC. B: The overexpression efficiency of oe-KLF2 confirmed by qRT-PCR in neuronal cells. C: The overexpression efficiency of oe-KLF2 determined by western blot analysis in neuronal cells. *, *p* < 0.05, *vs*. oe-NC treated neuronal cells. D: miR-26a expression as assessed by qRT-PCR in EVs and oe-KLF2 treated neuronal cells simultaneously. E: The expression of KLF9, TRAF2, and KLF2 determined by western blot analysis in neuronal cells treated with EVs-miR-26a inhibitor, oe-KLF2 or both. F: Apoptosis of neuronal cells treated with EVs-miR-26a inhibitor, oe-KLF2 or both examined by flow cytometry. G: The length of neurite outgrowth in neuronal cells treated with EVs-miR-26a inhibitor, oe-KLF2 or both analysed by Motic Images Plus. H: The expression of cleaved caspase-3, caspase-3, Bax, Bcl-2, NOGO-A, and NGF determined by western blot analysis in neuronal cells treated with EVs-miR-26a inhibitor, oe-KLF2 or both. I: Cerebral infarct area stained by TTC in brain tissue slices of mice treated with EVs-miR-26a inhibitor, oe-KLF2 or both. J: Identification of living neuronal cells by NeuN immunofluorescence staining in brain tissue slices of mice treated with EVs-miR-26a inhibitor, oe-KLF2 or both. K: Detection of cellular apoptosis by TUNEL staining in brain tissue slices of mice treated with EVs-miR-26a inhibitor, oe-KLF2 or both. In panels I-K, the dose of miR-26a inhibitor was 200 nM, and miR-26a inhibitor was injected into the mouse lateral ventricle together with the lentivirus expressing oe-KLF2. MCAO surgery was performed 3 days after the injection. After 72 h of reperfusion, the mice were euthanized for analysis. The dose of oe-KLF2 vector was 100 nM, and n = 8 for mice in each group [47]. *, *p* < 0.05, *vs*. EVs-inhibitor NC or oe-NC or EVs-inhibitor NC + oe-NC in mouse brain tissue slices, #, *p* < 0.05, *vs*. EVs-miR-26a inhibitor + oe-NC in mouse brain tissue slices. The measurement data were expressed as mean ± standard deviation. In panel A, B, C, and D, data were compared by unpaired *t-*test, in panel E, F, G, H, I, J, and K, data were compared by one-way ANOVA and Tukey’s post hoc test. The experiment was repeated three times independently.

The function of neuronal cells (Figure 6(f, G)) also indicated that compared with EVs-inhibitor NC + oe-NC, EVs-miR-26a inhibitor + oe-NC increased apoptosis and reduced neurite outgrowth after OGD/RP, while EVs-inhibitor-NC + oe-KLF2 inhibited apoptosis and increased the length of neurite outgrowth. miR-26a inhibitor + oe-KLF2 and EVs-miR-26a inhibitor + oe-NC inhibited apoptosis and increased the length of neurite outgrowth in neuronal cells. Western blot analysis (Figure 6h) also showed that, compared with EVs-inhibitor NC + oe-NC, EVs-miR-26a inhibitor + oe-NC increased expression of cleaved caspase-3/caspase-3, Bax and NOGO-A whereas reducing that of Bcl-2 and NGF, while EVs-inhibitor-NC + oe-KLF2 led to opposite results. Compared to EVs-miR-26a inhibitor + oe-NC, miR-26a inhibitor + oe-KLF2 inhibited the expression of cleaved caspase-3/caspase-3, Bax and NOGO-A, upregulated the expression of Bcl-2 and NGF and relieved neuronal damage in neuronal cells. Therefore, overexpression of KLF2 can alleviate neuronal damage caused by miR-26a inhibitor treatment.

To verify the role of KLF2 plays *in vivo*, we constructed a lentiviral overexpression vector (oe-KLF2). Oe-KLF2 and EVs were injected into the lateral ventricle of mice every 24 h for 3 days before MCAO surgery. After 72 h of reperfusion, the mice were euthanized and brain sections were taken to observe the area of cerebral infarction. EVs- miR-26a inhibitor + oe-KLF2 significantly reduced cerebral infarction area compared with EVs- miR-26a inhibitor + oe-NC in mouse brain tissue slices. TTC staining (Figure 6i) showed that compared with EVs-inhibitor-NC + oe-NC, EVs-inhibitor-NC + oe-KLF2 reduced cerebral infarct size, while EVs-miR-26a inhibitor + oe-NC increased the area of cerebral infarction. EVs-miR-26a inhibitor + oe-KLF2 significantly reduced the area of cerebral infarction compared to EVs-miR-26a inhibitor + oe-NC. The results of NeuN immunofluorescence staining (Figure 6j) and TUNEL staining (Figure 6k) of mouse brain tissue slices also revealed that, compared to EVs-inhibitor-NC + oe-NC, EVs-inhibitor-NC + oe-KLF2 reduced cell apoptosis and increased the number of NeuN + cells, while EVs-miR-26a inhibitor + oe-NC increased apoptosis and reduced the number of NeuN + cells. EVs-miR-26a inhibitor + oe-KLF2 significantly reduced apoptosis and increased the number of NeuN + cells in contrast to EVs-miR-26a inhibitor + oe-NC. Collectively these experiments clearly demonstrate that KLF2 overexpression also inhibit neuronal damage *in vivo*. Collectively, the results presented in this study indicate that ASC-EV-derived miR-26a inhibits neuronal damage *in vitro* and *in vivo* by regulating the KLF9/TRAF2/KLF2 regulatory axis.

## Discussion

The irreparable and irreversible nature of neuronal damage means that treatment following neuronal damage is often complex and unsuccessful. Therefore, research to alleviate neuronal damage is of significant therapeutic interest. EV-miRNAs have been shown to be overexpressed in cancers, leading to some specific miRNAs becoming the focus of work to identify novel biomarkers of disease [33–35]. Recent research has demonstrated that hsa-miR-1306-5p, hsa-miR-93-5p, hsa-miR-424-5p, and hsa-miR-3065-5p, and expression of P-S396-tau in EVs might provide a combinatorial protein and miRNA signature to differentiate between HC, patients with MCI or VaD from patient with sporadic AD [36]. Although EVs-miRNAs have been found to have a variety of functions, whether they play a role in neuronal injury and apoptosis remains unclear. Therefore, this study explores the role of ASC-EVs-miR-26a in neuronal cells, and showing that miR-26a in ASC-EVs can inhibit neuronal damage by regulating the expression axis of KLF9/TRAF2/KLF2. Evidence has indicated that EV-miRNAs could affect neuronal cells, and that hypoxia inducible factor-1α (HIF-1α)-miR-204-Bcl-2 pathway contributed to apoptosis of neuronal cells induced by hypoxia, which could potentially be exploited to prevent spinal cord ischaemia/reperfusion injury [37]. In addition, the types and abundances of miRNAs contained in exosomal cells from different sources are different. We found that the extracellular capsule of ASCs can reduce neuronal damage. Using a wide range of databases, we predicted that miR-26a in hair follicles can target KLF9. Studies have revealed that Expressing KLF7 or knocking out KLF4 in adult CNS neurons promotes axon regeneration after injury *in vivo* [38,39]. In some neuron subtypes, it has been found KLF9 has a number of functions including improving neuronal survival, increasing neurite growth, increasing the branching and elongation of neuronal cells, with the specific function depending on the level of its expression [40,41]. Nerve cells are highly sensitive to hypoxia, and apoptosis may occur under exposure to hypoxia.

**Figure 7.**
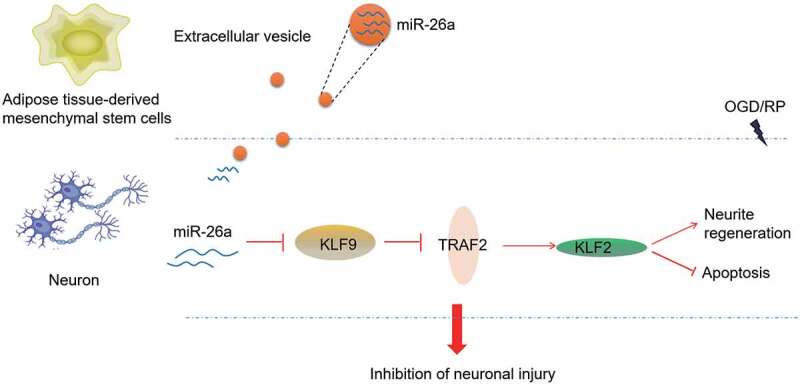
Schematic diagram summarizing the mechanism by which ASC-EVs-derived miR-26a acts during neuronal damage

Several apoptosis-related genes and miRNAs are involved in hypoxia-induced apoptosis [33]. Therefore, we further constructed cerebral ischaemia/reperfusion animal model and oxygen-glucose deprivation/reperfusion cell model. ASC-EVs slow the damage to neuronal cells by cerebral ischaemia/reperfusion. We extracted miRNAs from ASC-EVs and demonstrated that miR-26a secreted could be transferred to neuronal cells to alleviate their damage. At present, there is no clear literature showing that miR-26a can alleviate neuronal damage, but studies have confirmed that miR-26a can target interleukin-6 (IL-6) [42] and transient receptor potential canonical 3 (TRPC3) [43]. Therefore, we predicted miR-26a and neuronal cell injury-related factors through online-related websites, and found that KLF9 may play an important role in neuronal injury. We further clarified that miR-26a can target KLF9 and that miR-26a mimic can significantly inhibit KLF9 expression through a dual-luciferase reporter test. The effect of miR-26a was reversed by overexpression of KLF9. ChIP-seq data revealed that KLF9 can bind to the TRAF2 promoter, and previous data have shown that TRAF2 can inhibit neuronal damage after MCAO [44]. Inhibition of Sphk1 with inhibitors or small interfering RNA (siRNA) in microglial cells can reduce the expression of TRAF2 and nuclear factor-κB (NF-κB), and the ensuing neuronal apoptosis in response to OGD/RP was also arrested [45]. Moreover, the ischaemic damage of neuronal cells increases the cytotoxicity and apoptosis of cells, and is accompanied by the continuous activation of IRE1-alpha/TRAF2, JNK1/2, and p38 MAPK pathways [46]. We report that KLF9 can regulate the expression of TRAF2 and KLF2, and that KLF9 may affect neuronal damage through TRAF2 and KLF2. *In vivo* and *in vitro* experiments, by overexpressing KLF2 and co-processing neuronal cells with EVs, we showed that overexpression of KLF2 can alleviate neuronal damage caused by miR-26a inhibitor treatment,

In summary, the findings in the current study demonstrate that ASC-EVs transfer miR-26a to neuronal cells where miR-26a targets and inhibits KLF9 expression, and thus relieves the inhibition of TRAF2/KLF2 axis by KLF9, eventually arresting neuronal damage (Figure 7). These novel findings might provide a potential treatment for neuronal damage in the future. Due to the limited data supporting the targeting between miR-26a and KLF9, our subsequent endeavours are still required to fully realize the potential of this new mechanism.

ASC-EVs contain a large amount of miR-26a and transfer miR-26a into neuronal cells. miR-26a targets and inhibits the expression of KLF9, which relieves the transcriptional inhibition of TRAF2 by KLF9. This increases the expression of TRAF2, increasing the expression of KLF2 and thereby inhibiting neuronal damage.

## Supplementary Material

Supplemental Material

Supplemental Material

Supplemental Material

## Data Availability

The datasets generated and analysed during the current study are available.
