# Supplementary material for: microRNA-26a shuttled by extracellular vesicles secreted from adipose-derived mesenchymal stem cells reduce neuronal damage through KLF9-mediated regulation of TRAF2/KLF2 axis
Source: Adipocyte. 2021 Jul 26;10(1):378–93. doi: 10.1080/21623945.2021.1938829 (PMC8320674; doi:10.1080/21623945.2021.1938829)
Supplement: Supplemental Material [file KADI_A_1938829_SM4062.docx]

**Supplementary Figure 1** Analysis of ASCs and EVs.

A: FACS analysis of ASCs. Isotype control is marked in orange. The detection of the expression of ASC surface molecular markers is shown by the green line. B: DLS analysis of EV diameter. C: FACS analysis and statistical histogram of MAP2 detected by immunofluorescence in mouse primary cortical neuron cells. Isotype control is marked in orange. The detection of the expression of surface molecular markers is shown by the green line. D: Western blot analysis of EV intake in the brain tissues. n = 8 for mice in each group.
