# Supplementary figures and images for: microRNA-26a shuttled by extracellular vesicles secreted from adipose-derived mesenchymal stem cells reduce neuronal damage through KLF9-mediated regulation of TRAF2/KLF2 axis
Source: Adipocyte. 2021 Jul 26;10(1):378–93. doi: 10.1080/21623945.2021.1938829 (PMC8320674; doi:10.1080/21623945.2021.1938829)

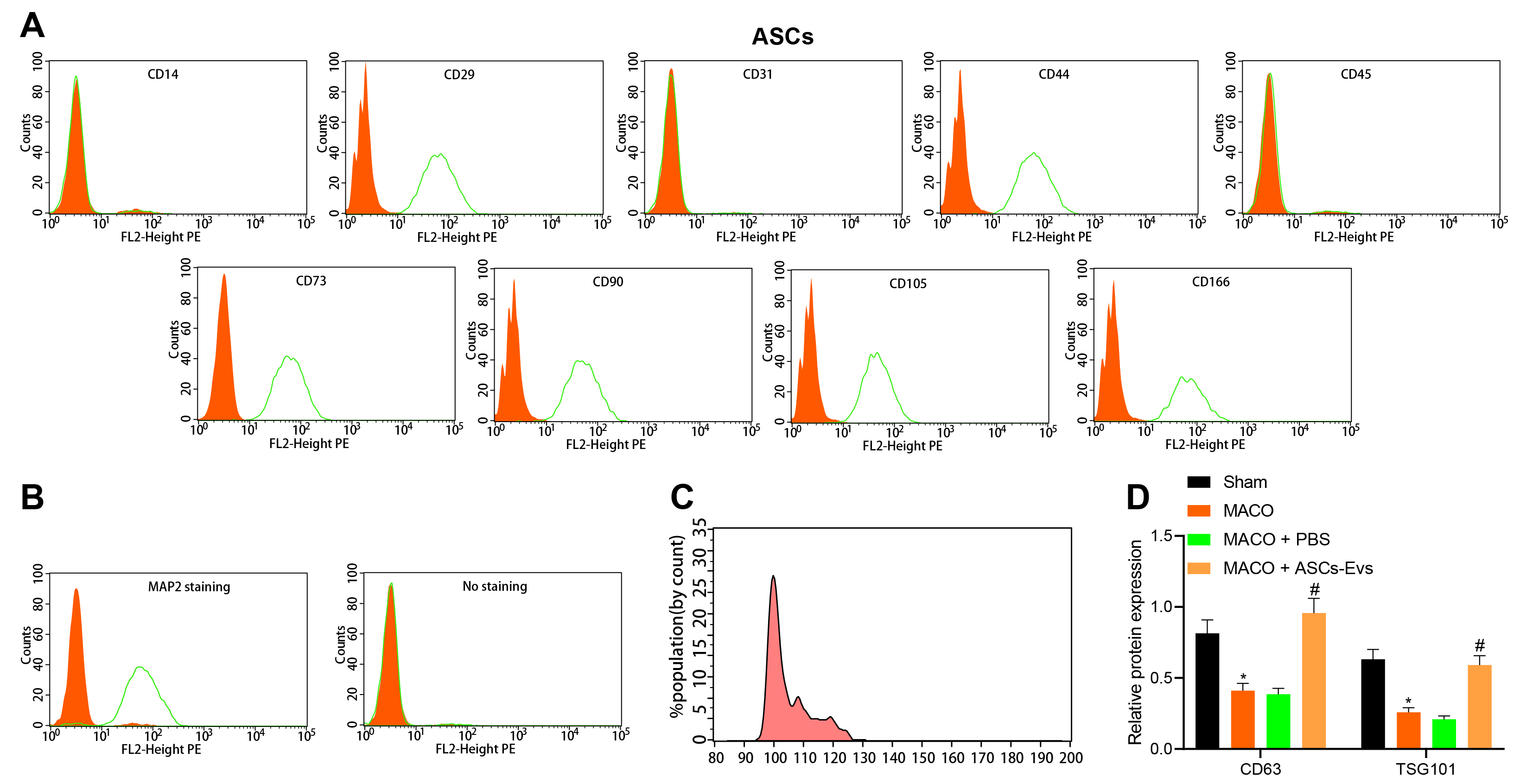

Supplement: Supplemental Material [file KADI_A_1938829_SM4059.jpg]
