# Supplementary material for: microRNA-26a shuttled by extracellular vesicles secreted from adipose-derived mesenchymal stem cells reduce neuronal damage through KLF9-mediated regulation of TRAF2/KLF2 axis
Source: Adipocyte. 2021 Jul 26;10(1):378–93. doi: 10.1080/21623945.2021.1938829 (PMC8320674; doi:10.1080/21623945.2021.1938829)
Supplement: Supplemental Material [file KADI_A_1938829_SM4057.docx]

**Supplementary Table 1** Primer sequences for qRT-PCR

| Target | Forward sequence (5′-3′) | Reverse sequence (5′-3′) |
| --- | --- | --- |
| miR-26a | TCGCCGTTCAAGTAATCCAG | CAGAGCAGGGTCCGAGGTA |
| Cel-miR-39 | UCACCGGGUGUAAAUCAGCUUG | CAGAGCAGGGTCCGAGGTA |
| KLF9 | GCCGCCTACATGGACTTCG | GGTCACCGTGTTCCTTGGT |
| TRAF2 | AGAGAGTAGTTCGGCCTTTCC | GTGCATCCATCATTGGGACAG |
| KLF2 | GAGCCTATCTTGCCGTCCTTT | CACGTTGTTTAGGTCCTCATCC |
| GAPDH | AGGTCGGTGTGAACGGATTTG | GGGGTCGTTGATGGCAACA |
